# Ordinal Preferential Attachment: A Self-Organizing Principle Generating Dense Scale-Free Networks

**DOI:** 10.1038/s41598-019-40716-1

**Published:** 2019-03-11

**Authors:** Taichi Haruna, Yukio-Pegio Gunji

**Affiliations:** 1 Department of Information and Sciences, School of Arts and Sciences, Tokyo Woman’s Christian University, 2-6-1 Zempukuji, Suginami-ku, Tokyo, 167-8585 Japan; 2 Department of Intermedia Art and Science, School of Fundamental Science and Technology, Waseda University, 3-4-1 Ohkubo, Shinjuku-ku, Tokyo, 169-8555 Japan

## Abstract

Networks are useful representations for analyzing and modeling real-world complex systems. They are often both scale-free and dense: their degree distribution follows a power-law and their average degree grows over time. So far, it has been argued that producing such networks is difficult without externally imposing a suitable cutoff for the scale-free regime. Here, we propose a new growing network model that produces dense scale-free networks with dynamically generated cutoffs. The link formation rule is based on a weak form of preferential attachment depending only on order relations between the degrees of nodes. By this mechanism, our model yields scale-free networks whose scaling exponents can take arbitrary values greater than 1. In particular, the resulting networks are dense when scaling exponents are 2 or less. We analytically study network properties such as the degree distribution, the degree correlation function, and the local clustering coefficient. All analytical calculations are in good agreement with numerical simulations. These results show that both sparse and dense scale-free networks can emerge through the same self-organizing process.

## Introduction

Complex systems in nature and society can be represented as networks^1–3^. A ubiquitous feature of networks is that they are scale-free^4^, meaning their degree distribution follows a power-law ${p}_{k}\simeq c{k}^{-\gamma }$, where *k* denotes the number of neighbors a node has (the *degree*), *p*_*k*_ is the fraction of nodes with degree *k*, and $\simeq$ indicates asymptotic equality for large *k*. Over the last two decades, sparse scale-free networks with $2 < \gamma  < 3$ have attracted a lot of attention^5–7^. The most popular mechanism for producing such networks is growth and preferential attachment^4^, in which existing nodes acquire links from new nodes with a probability proportional to their degree. Indeed, the Barabási–Albert model^4^ and variants have successfully generated scale-free networks with $2 < \gamma$ by considering initial node attractiveness^8^, nonlinearity of preferential attachment^9^, and node fitness^10^, to name but a few.

Recently, there is increasing interest in dense scale-free networks with exponents $\gamma \le 2$, because such networks are often found in social, informational and molecular networks^11–15^. Networks are called dense when their average degree diverges as they grow. However, generating dense scale-free networks is difficult without applying external constraints^16^. Although there are a few models that can generate dense scale-free networks with a particular *γ* value ($\gamma =2$^11,17,18^, $\tfrac{3}{2}$^12^), to the authors’ knowledge, the only models with adjustable *γ* is a configuration model with externally given explicit cutoffs for the scale-free regime^12^. Some authors have proposed network models that can generate networks whose degree distribution follows a power-law with exponent $1\le \gamma \le 2$ up to a constant degree and decays exponentially beyond that^15,19,20^. However, we do not call these networks dense, since they have a finite average degree in the limit of large network size.

In this paper, we propose a growing network model with a single parameter *δ* that can arbitrarily control the exponent *γ* in the range $\gamma  > 1$ without externally imposing cutoffs. Our model copies a node with its degree regarded as its ability to form links. Parameter *δ* is the conversion coefficient from the actual number of links a node has to the node’s ability to form links. New links between a new node and existing nodes are formed following a rule called *ordinal preferential attachment*, by which the new node connects to existing nodes having higher ability to form links than itself. We find the probability that an existing node acquires a new link to be proportional to its degree in a range up to an order of *t*^1/*γ*^, where *t* is the number of nodes in the network. This leads to an internally generated cutoff for the scale-free regime and makes it possible to yield dense scale-free networks when $1\le \delta  < e$, as we show below.

## Model

Our model assumes time-scale separation between node addition and link formation, as in the case of conventional growing network models. We also assume that every node has at least one link. When the initial network satisfies this assumption, it also holds for all time steps in the network evolution algorithm presented below. Each time step repeats the following two procedures:

### Copying Degree

An existing node *y* is chosen uniformly at random. A new node *x* together with its *virtual degree* ${d}_{x}^{\ast }$ is generated by incompletely copying the degree *d*_*y*_ of the chosen node *y* with a scale factor $\delta  > 0$. Namely, ${d}_{x}^{\ast }$ is chosen uniformly at random from the set of integers between 1 and $\lceil \delta {d}_{y}\rceil$, where $\lceil a\rceil$ is the smallest integer greater than or equal to *a*.

### Ordinal Preferential Attachment

Let *n* be the number of existing nodes *z* satisfying

1 $${d}_{x}^{\ast }\le \lceil \delta {d}_{z}\rceil ,$$

where *d*_*z*_ is the degree of *z*. If ${d}_{x}^{\ast }\le n$, the new node *x* forms links with ${d}_{x}^{\ast }$ randomly chosen nodes *z*. Otherwise, *x* is connected to all nodes *z* satisfying Eq. (1). The actual degree of *x* is ${d}_{x}={d}_{x}^{\ast }$ in the former case, while ${d}_{x}=n$ in the latter case.

**Figure 1:**
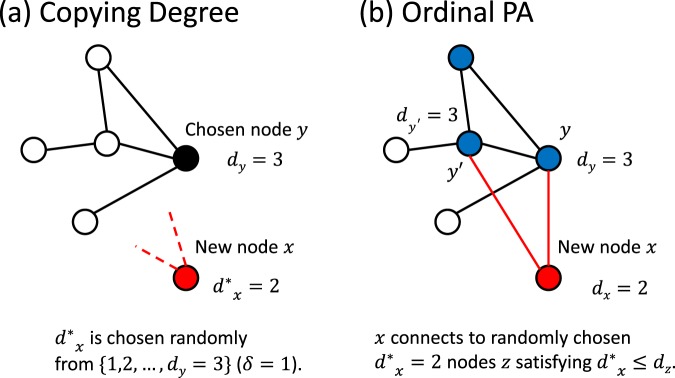
The link formation process in our model with $\delta =1$. (**a**) Copying degree. Node *y* is chosen and its degree ${d}_{y}=3$ is copied. A new node *x* is generated with virtual degree ${d}_{x}^{\ast }=2\le 3$. (**b**) Ordinal preferential attachment. There are three nodes with degree greater than or equal to ${d}_{x}^{\ast }=2$ (colored blue). Among them, *y* and *y*′ are chosen as targets of links emanating from *x*. As a result, *d*_*x*_ is determined to be 2.

The scale factor *δ* regulates comparisons between the ability of a new node to form links, represented as its virtual degree, and the actual number of links (degree) of an existing node. In Fig. 1, the algorithm in our model when $\delta =1$ is illustrated.

The above link formation rule is motivated by the following considerations. By definition, the degree of each node is just the number of adjacent nodes resulting from the link formation process. However, the degree of a node can act as a cause to form links as assumed in the conventional preferential attachment models (the “popularity is attractive” principle^21^). Our first idea is to incorporate this principle in the copying process. Specifically, ‘popularity’—the ability to form links—is copied to new nodes. The second idea is to represent preferential attachment from the perspective of the new node. Specifically, the new node connects to more ‘popular’ existing nodes *than itself*. We give a further interpretation in the Discussion section.

Our model assumes that the new node can know the degree of every existing node. In other words, the new node has enough time to examine the degree of all existing nodes before another new node is created, which is consistent with the time-scale separation between node addition and link formation. This assumption differs from that in conventional preferential attachment using the degree distribution^4^ and that in copying models using local link information^11,22–25^.

Note that we allow the case where $\delta  > 1$. Thus, newly added nodes may have a larger virtual degree than the degree of the copied node. As we will see, this makes it possible to produce dense scale-free networks. In the following numerical simulations, the initial network consists of two nodes and a link between them. If the network evolution begins with a connected network with 2 or more nodes, then the evolved network is connected at all time steps thereafter, since ${d}_{x}^{\ast }\ge 1$ and there is at least one node *z* satisfying Eq. (1), namely, node *y*. For simplicity, we identify time steps in the network evolution with the number of nodes.

## Results

Let *p*_*k*_(*t*) be the fraction of nodes with degree *k* at time step *t*. For $k > 0$, *p*_*k*_(*t*) follows the rate equation

2 $$(t+1){p}_{k}(t+1)=t{p}_{k}(t)+{a}_{k-1}(t)t{p}_{k-1}(t)-{a}_{k}(t)t{p}_{k}(t)+{b}_{k}(t),$$

where *a*_*k*_(*t*) is the probability that an existing node with degree *k* acquires a link at time step *t*, and *b*_*k*_(*t*) is the probability that a node newly added at time step *t* has degree *k*. Let *q*_*k*_(*t*) be the probability that the virtual degree of the node newly added at time step *t* is *k*, which is given by

3 $${q}_{k}(t)=\sum _{k\le \lceil \delta l\rceil  < t}\,{p}_{l}(t)\frac{1}{\lceil \delta l\rceil }.$$

We show that Eq. (2) has a power-law solution ${p}_{k}\simeq c{k}^{-\gamma }$ for $t\gg 1$ and $1\ll k < {k}^{\ast }(t)$, where ${k}^{\ast }(t)\sim {t}^{1/\gamma }$ (~ indicates asymptotic proportionality for large *t*), and the exponent *γ* is determined by a self-consistent argument. Assume that ${p}_{k}=c{k}^{-\gamma }$ with $\gamma  > 1$ and that *p*_*k*_(*t*) does not decay slower than *k*^−*γ*^ for $k\gg {k}^{\ast }(t)$. As described in the Methods section, it can be shown that

4 $${b}_{k}(t)={q}_{k}(t)\simeq \frac{c{\delta }^{\gamma -1}}{\gamma }{k}^{-\gamma }$$

and

5 $${a}_{k}(t)\simeq \frac{\gamma -1}{\gamma t}\delta k$$

for $1\ll k < {k}^{\ast }(t)$. For $k\gg {k}^{\ast }(t)$, *a*_*k*_(*t*) is a constant as a function of *k* and it follows that *p*_*k*_ decays exponentially. Thus, the mechanism of preferential attachment itself grows as the network evolves. For $1\ll k < {k}^{\ast }(t)$, the continuous approximation of Eq. (2) for $t\gg 1$ leads to

6 $$\frac{\partial ({a}_{k}t{p}_{k})}{\partial k}={q}_{k}-{p}_{k}.$$

By comparing the coefficients of *k*^−*γ*^ on the both sides of Eq. (6), we obtain the following equation that *γ* must satisfy:

7 $$\gamma =\delta {(\gamma -1)}^{2}+{\delta }^{\gamma -1}.$$

**Figure 2:**
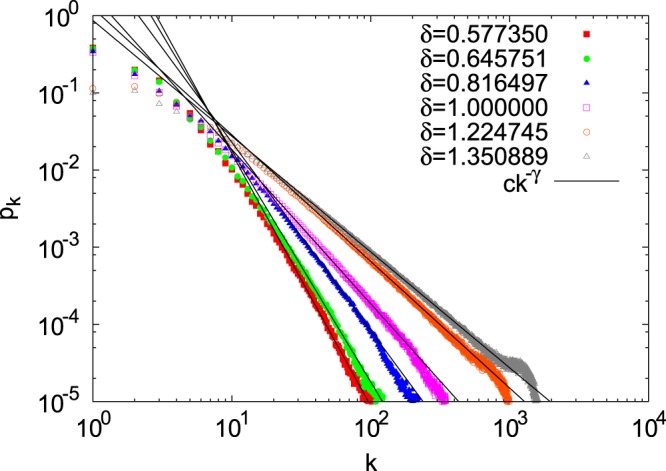
Degree distributions at time step $t=65536$. Solid lines are *ck*^−*γ*^ where *γ* is obtained from Eq. (7) and *c* is determined by fitting the functional form to the numerical data. The values of *γ* are: $\gamma =3.275172\ldots$ for $\delta =\frac{1}{\sqrt{3}}=0.577350\ldots$, $\gamma =3$ for $\delta =-\,2+\sqrt{7}=0.643751\ldots$, $\gamma =2.440080\ldots$ for $\delta =\sqrt{\frac{2}{3}}=0.816497\ldots$, $\gamma =2$ for $\delta =1$, $\gamma =1.639752\ldots$ for $\delta =\sqrt{\frac{3}{2}}=1.224745\ldots$, and $\gamma =\frac{3}{2}$ for $\delta =14-4\sqrt{10}=1.350889\ldots$.

For all $\delta  > 0$, $\gamma =1$ is a solution of Eq. (7). However, we ignore this solution since *p*_*k*_ cannot be normalized when $\gamma =1$. Equation (7) has another solution *γ* when $0 < \delta  < e=2.718281\ldots$. *γ* approaches 1 as $\delta \to e$ and diverges as $\delta \to 0$. In Fig. 2, the numerically obtained degree distributions at $t=65536$ for six values of *δ* are shown together with power-law degree distributions ${p}_{k}=c{k}^{-\gamma }$, where *γ* is a solution of Eq. (7) such that $\gamma \ne 1$ and *c* is determined by fitting distribution tails. The analytically determined exponents *γ* agree well with the numerical simulations.

It has been shown that scale-free networks with exponent $\gamma \le 2$ must have a cutoff^16^. In our model, the power-law regime has cutoff ${k}^{\ast }(t)\sim {t}^{1/\gamma }$ (see the first subsection in the Methods section) and k*(*t*) emerges as a consequence of the network growth mechanism, rather than being externally given. The range is narrower than that for conventional sparse networks of order *t*^1/(*γ*−1)^^3^. However, the probability that a node has a degree larger than k*(*t*) approaches 0 as $t\to \infty$ under the assumption that *p*_*k*_ does not decay slower than *k*^−*γ*^ for $k > {k}^{\ast }(t)$.

In the following, we investigate a few basic properties of the networks generated by our model. Let *k*_*s*_(*t*) be the average degree of a node at time step *t* that is newly added at time step *s* with degree ${k}_{s}(s)=d$. It then follows that

8 $$\frac{d{k}_{s}}{dt}={a}_{{k}_{s}}(t)\simeq \frac{(\gamma -1)\delta }{\gamma t}{k}_{s}$$

for all $1\ll {k}_{s} < {k}^{\ast }(t)$ and $t\gg 1$. If $1\ll {k}_{s}(t) < {k}^{\ast }(t)$, this is solved by

9 $${k}_{s}(t)=d{(\frac{t}{s})}^{\beta },$$

where $$\beta =\frac{\gamma -1}{\gamma }\delta $$.

By making use of *k*_*s*_(*t*), we can place analytical lower bounds on the degree correlation function^26^ *k*_nn_(*k*) and the local clustering coefficient^27^ *C*(*k*). First, consider *k*_nn_(*k*) defined as the average degree of the neighbors of a node with degree *k*. By considering fluctuation of the initial degree *k*_*s*_(*s*) unlike the conventional mean-field calculations^28^, we obtain (see the Methods section)

10 $${k}_{{\rm{nn}}}(k)\ge \frac{\delta }{2}k$$

for $1\ll k < {k}^{\ast }(t)$. *C*(*k*), defined as the probability that a pair of nodes among neighbors of a degree-*k* node is connected, can be treated similarly in a moderately dense regime. Indeed, when −1 + *β*(*γ* + 2) > 0 (equivalently, either $\delta  < 1.796028\ldots$ or $\gamma  > 1.209863\ldots$), we have (see the Methods section)

11 $$C(k)\ge \frac{D}{t}{k}^{\gamma }$$

for $$1\ll k < {k}^{\ast }(t)$$, where

12 $$D=\frac{2{\delta }^{\gamma +3}{(\gamma -1)}^{2}}{c(\gamma +2)(\gamma +1)\gamma (\,-\,1+\beta (\gamma +2))}.$$

**Figure 3:**
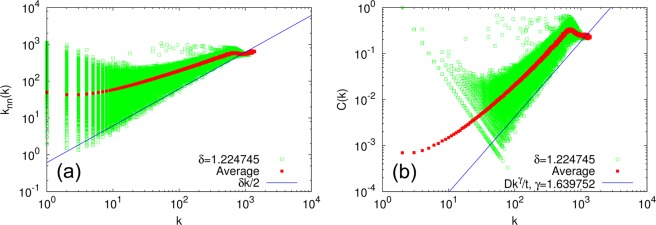
(**a**) Degree correlation function *k*_nn_(*k*) and (**b**) local clustering coefficient *C*(*k*) for $\delta =\sqrt{\frac{3}{2}}=1.224745\ldots$. Open squares are single-trial results, each representing the corresponding quantity for a single node at time step $t=65536$. Filled squares are averages over 100 trials. Solid lines are analytical lower bounds: (**a**) $\frac{\delta }{2}k$, and (**b**) $\frac{D}{t}{k}^{\gamma }$ with $\gamma =1.639752\ldots$.

Figure 3 compares the analytical lower bounds Eqs (10) and (11) with numerical simulations for $$\delta =\sqrt{\frac{3}{2}}=$$$$1.224745\ldots $$ (Fig. 3(a and b), respectively), showing good agreement between the analytical lines and numerical results.

Equation (11) can be used to evaluate the behavior of the average local clustering coefficient^29^ *C* for $t\gg 1$ when $\delta  < 1.796028\ldots$:

13 $$C=\int \,dk\,{p}_{k}C(k)\ge {\int }^{{k}^{\ast }}\,dk\,c{k}^{-\gamma }\frac{D}{t}{k}^{\gamma }\sim {t}^{\frac{1-\gamma }{\gamma }}.$$

**Figure 4:**
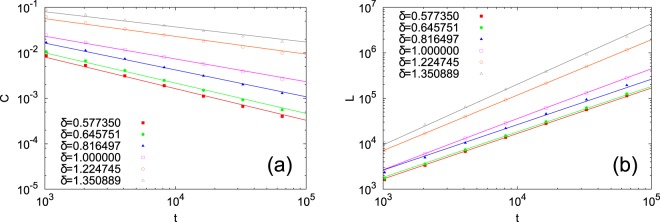
(**a**) The average clustering coefficient averaged over 100 trials as a function of *t*. Solid lines are best fits to the right side of Eq. (13). (**b**) The number of links averaged over 100 trials as a function of *t*. Solid lines are best fits to Eq. (14).

Figure 4(a) suggests that scaling of the lower bound on *C* in Eq. (13) approximates that of *C*. Contrary to uncorrelated dense scale-free networks with $C > 0$^12^, our numerical simulations indicate that *C* vanishes as $t\to \infty$ in our model, due to the strong positive correlation between the degrees of neighboring nodes, as shown in Fig. 3(a). However, it follows that the evolved networks are more clustered than in classical Erdös-Rényi random networks with the same average degree. This can be immediately deduced from Eq. (14) below and the fact that the average local clustering coefficient of the latter is equal to the probability that there is a link between two randomly chosen nodes^1^.

The scaling of the number of links *L* is obtained as

14 $$L=\frac{t}{2}\langle k\rangle \simeq {\int }^{{k}^{\ast }}\,dk\,c{k}^{-\gamma +1}\sim \{\begin{array}{ll}t & (\gamma  > 2)\\ t\,\mathrm{ln}\,t & (\gamma =2)\\ {t}^{2/\gamma } & (1 < \gamma  < 2)\end{array}.$$

Figure 4(b) confirms that the scaling of Eq. (14) agrees well with the numerical simulations. *L* diverges faster than *t* when $\gamma \le 2$, confirming that the evolved networks are actually dense. The above agreements between analytical calculations and numerical results for properties of the evolved networks support our self-consistent argument for determining power-law exponents by Eq. (7).

## Discussion

The analytical lower bounds on *k*_nn_(*k*) by Eq. (10) and the numerical results in Fig. 3(a) indicate that the evolved networks in our model are strongly assortative^30^ for large *k*. The analytical lower bound for *C*(*k*) by Eq. (11) and the numerical results in Fig. 3(b) together suggest that higher-degree nodes tend to connect to nodes with similar degrees and to form triangles. We also note that by Eq. (1), a new node with small virtual degree can connect to large-degree nodes. This results in large variance in data from a single trial in Fig. 3. Thus, networks evolved by our model have a core–periphery structure like those observed in real-world networks^31^. Note that real-world networks rarely have an explicit scaling in the form of *C*(*k*) with a positive exponent^27^. However, such a discrepancy with real-world data in higher-order network properties can be overcome by introducing additional link formation, deletion, or rewiring mechanisms that do not affect the degree distribution. Such modifications are not hard, because ordinal preferential attachment depends only on node degrees.

The following compares our proposed model with previous attempts to generate dense scale-free networks, mentioned in the Introduction. Bhat *et al*.^17^ considered a growing network model based on link copying. In this model, nodes are newly created by randomly choosing an existing node and copying each link from the chosen node to its neighbors with independent probability *p*. A link between the new node and the chosen node is also made. The model studied in Ispolatov *et al*.^11^ is similar, except that no additional link between the new and chosen nodes is created. Here, we focus on the model by Bhat *et al*., which was shown to generate sparse scale-free networks with exponents $\gamma  > 2$ for $p < \frac{1}{2}$, while generated networks are dense when $p\ge \frac{1}{2}$. In the dense regime, power-law degree distributions are allowed only when $p=\frac{1}{2}$ with $\gamma =2$. The authors showed that when $p > \frac{1}{2}$, the degree distributions are no longer scale-free and have a peak whose location diverges as the networks grow. This is due to strong history dependence of network growth, as indicated by the non-self-averaging property of the number of links for $p > \frac{1}{2}$. Recall that our model copies the degree of the chosen node when adding a new node. Such a weaker copying process could inhibit large fluctuations between generated networks by individual trials and is consistent with dense scale-free networks. Courtney and Bianconi^18^ proposed a framework for generating dense scale-free networks based a stochastic process called the Pitman–Yor process, which can produce power-law distributions with exponents $1 < \gamma \le 2$. This model allows the existence of multiple links for nodes pairs. In this case, the number of links emanating from a node is called its *strength*. The link formation process depends on a form of preferential attachment using the strength distribution. Courtney and Bianconi showed that the strength distribution of generated networks follows a power-law with an exponent $1 < \gamma \le 2$. However, they also showed that the degree distribution collapses to a power-law distribution with $\gamma =2$. This work suggests the difficulty of designing growing network models generating dense scale-free networks from general stochastic processes that are not intended to model network evolution. Seyed-Allaei *et al*.^12^ showed that the combination of random addition of a new node with merging of two randomly chosen nodes yields a dense scale-free network with $\gamma =\frac{3}{2}$. However, no known extension of this model can generate dense scale-free networks with exponents other than $\frac{3}{2}$.

Finally, we discuss the logic of link formation in the proposed model. First, the virtual degree of a new node *x* is determined so that *there exists* a node *y* such that ${d}_{x}^{\ast }\le \lceil \delta {d}_{y}\rceil$. Second, the set of neighbors of *x* is determined so that ${d}_{x}^{\ast }\le \lceil \delta {d}_{z}\rceil$ *for all* neighbors *z*. Here, a substitution of the quantifiers occurs. Namely, the universal quantifier “for all” in the second step is substituted for the existential quantifier “there exists” in the first step. This can be interpreted as introducing the endo-perspective^32^ to the friendship paradox^33^ for friendship networks. Conventionally, the reason why individuals often feel that their friends have more friends than they do is ascribed to elementary network arithmetic. Namely, this is due to the difference between node degrees and degrees of neighboring nodes. However, the paradox could motivate link formation from each individual’s perspective. Indeed, in our model, the paradox is once invalidated for a new node *x* by forgetting the neighbors of the original node *y*, then used as a driving force of link formation; the new node *x* tries to form links with more ‘popular’ existing nodes than itself. Such openness of interpretation in terms of individual perspectives constitutes the endo-perspective in the friendship paradox. It compensates for the lack of link information in the link formation process, leading to growing preferential attachment. This in turn yields the power-law degree distributions ${p}_{k}\simeq c{k}^{-\gamma }$ with a cutoff on the order of *t*^1/*γ*^ with $\gamma  > 1$. This work demonstrates the efficacy of mathematical modeling of complex systems by incorporating the endo-perspective.

## Methods

### Derivations of Eqs (4) and (5)

Let *N*_*k*_(*t*) denote the number of nodes whose degree *l* satisfies $k\le \lceil \delta l\rceil$. Namely,

15 $${N}_{k}(t)=t\,\sum _{k\le \lceil \delta l\rceil  < t}\,{p}_{l}(t).$$

By using *q*_*k*_(*t*) and *N*_*k*_(*t*), *a*_*k*_(*t*) and *b*_*k*_(*t*) can be respectively expressed as

16 $${a}_{k}(t)=\sum _{l=1}^{\lceil \delta k\rceil }\,{q}_{l}(t)(\theta ({N}_{l}(t)-l)\frac{l}{{N}_{l}(t)}+(1-\theta ({N}_{l}(t)-l)))$$

and

17 $${b}_{k}(t)={q}_{k}(t)\theta ({N}_{k}(t)-k)+\sum _{l=k+1}^{t-1}\,{q}_{l}(t){\delta }_{{N}_{l}(t),k},$$

where *θ* is a step function such that $\theta (x)=1$ if $x\ge 0$ and otherwise $\theta (x)=0$, and *δ*_*i*,*j*_ is the Kronecker delta.

Assume that ${p}_{k}=c{k}^{-\gamma }$ for $t\gg 1$ and $k\gg 1$ where $\gamma  > 1$. Further, suppose that *p*_*k*_(*t*) does not decay slower than *k*^−*γ*^ for *k* satisfying ${N}_{k}(t)\le k$. We mainly focus on the range of *k* where ${N}_{k}(t) > k$ holds. Since

18 $${N}_{k}(t)\simeq t\,{\int }_{k/\delta }^{\infty }\,dl\,{p}_{l}(t)\le t\,{\int }_{k/\delta }^{\infty }\,dl\,c{l}^{-\gamma }=t\frac{c}{\gamma -1}{(\frac{k}{\delta })}^{-\gamma +1},$$

a necessary condition for ${N}_{k}(t) > k$ is

19 $$k < M{t}^{1/\gamma },$$

where $M={(\frac{c}{(\gamma -1){\delta }^{-\gamma +1}})}^{1/\gamma }$. In the following, we assume that ${N}_{k}(t) > k$ holds for $1\ll k < M^{\prime} {t}^{1/\gamma }$, where *M*′ is a constant such that $0 < M^{\prime}  < M$. Consequently, for $1\ll k < {k}^{\ast }(t)\,:\,=M^{\prime} {t}^{1/\gamma }$, Eqs (16) and (17) respectively reduce to

20 $${a}_{k}(t)=\sum _{l=1}^{\lceil \delta k\rceil }\,{q}_{l}(t)\frac{l}{{N}_{l}(t)}$$

and

21 $${b}_{k}(t)={q}_{k}(t).$$

Since *q*_*k*_(*t*) can be approximated as

22 $${q}_{k}(t)\simeq {\int }_{k/\delta }^{\infty }\,dl\,c{l}^{-\gamma }\frac{1}{\delta l}=\frac{c{\delta }^{\gamma -1}}{\gamma }{k}^{-\gamma }$$

for $1\ll k < {k}^{\ast }(t)$, we obtain Eq. (4). Furthermore, we have ${N}_{k}(t)\simeq t\frac{c}{\gamma -1}{(\frac{k}{\delta })}^{-\gamma +1}$ in the leading order of *t*. It follows that each term with $l\gg 1$ on the right side of Eq. (20) can be approximated as

23 $${q}_{l}(t)\frac{l}{{N}_{l}(t)}\simeq \frac{c{\delta }^{\gamma -1}}{\gamma }{l}^{-\gamma }\frac{l}{t\frac{c}{\gamma -1}{(\frac{l}{\delta })}^{-\gamma +1}}=\frac{\gamma -1}{\gamma t}.$$

By substituting Eq. (23) into Eq. (20), we obtain Eq. (5), which was

$${a}_{k}(t)\simeq \frac{\gamma -1}{\gamma t}\delta k$$

for $$1\ll k < {k}^{\ast }(t)$$.

### Derivation of Eq. (10)

We begin by calculating *k*_nn_(*k*) as a function of three variables: time step *s* when the focal node *x* is added, its initial degree ${k}_{s}(s)=d$, and time step *t* when the average degree of its neighbors is calculated. Neighbors of *x* can be divided into two groups. The first group consists of those added after time step *s*. The members of the second groups are those added before time step *s*. In the following, we consider only the first group to derive the lower bound on *k*_nn_(*k*). Let $s\ll t$. We consider *d* such that ${\rm{\max }}\,\{1,\delta \}{k}_{s}(s^{\prime} ) < {k}^{\ast }(s^{\prime} )\sim {s^{\prime} }^{1/\gamma }$ holds for all $s\le s^{\prime} \le t$. Those not satisfying this condition can be ignored because their contribution to the degree distribution *p*_*k*_ at time step *t* approaches 0 as $t\to \infty$ under the assumption that *p*_*k*_ does not decay slower than *k*^−*γ*^ for $k > {k}^{\ast }(t)$. Then, we have

24 $$\begin{array}{ccc}{k}_{{\rm{n}}{\rm{n}}}(s,d,t) & \ge  & \frac{1}{{k}_{s}(t)}\,\sum _{s < s^{\prime}  < t}\,\sum _{k\le \lceil \delta {k}_{s}(s^{\prime} )\rceil }\,{q}_{k}\frac{k}{{N}_{k}(s{\rm{^{\prime} }})}{k}_{s{\rm{^{\prime} }}}(t)\\  & \simeq  & \frac{1}{d{(\frac{t}{s})}^{\beta }}\,{\int }_{s}^{t}\,ds{\rm{^{\prime} }}\,{\int }^{\delta d{(\frac{s{\rm{^{\prime} }}}{s})}^{\beta }}\,dk\,\frac{\gamma -1}{\gamma s{\rm{^{\prime} }}}k{(\frac{t}{s{\rm{^{\prime} }}})}^{\beta }\\  & = & \frac{\delta }{2}d({(\frac{t}{s})}^{\beta }-1)\\  & \simeq  & \frac{\delta }{2}{k}_{s}(t),\end{array}$$

where $1\ll {k}_{s}(t) < {k}^{\ast }(t)$, and we set $k={k}_{s^{\prime} }(s^{\prime} )$. Since the last expression in Eq. (24) is a function of *k*, we can write

$${k}_{{\rm{nn}}}(k)\ge \frac{\delta }{2}k.$$

### Derivation of Eq. (11)

We can similarly calculate a lower bound on *C*(*k*). Given a node *x* added at time step *s* with degree *d*, we measure its local clustering coefficient at time step *t* as the probability that a pair of its neighbors is connected. When calculating the lower bound, we consider only the neighbors that are added after time step *s*. As in the case of Eq. (24), we can focus on *d* such that ${\rm{\max }}\,\{1,{\delta }^{2}\}{k}_{s}(s^{\prime} ) < {k}^{\ast }(s^{\prime} )$ holds for all $s\le s^{\prime} \le t$ for given $s\ll t$. When −1 + *β*(*γ* + 2) > 0 (equivalently, either $\delta  < 1.796028\ldots$ or $\gamma  > 1.209863\ldots$), we have

25 $$\begin{array}{ccc}C(s,d,t) & \ge  & {\textstyle \tfrac{2}{{k}_{s}(t)\,({k}_{s}(t)-1)}}\,\sum _{s < s{\rm{^{\prime} }} < t}\,\sum _{s{\rm{^{\prime} }} < s{\rm{^{\prime} }}{\rm{^{\prime} }} < t}\,\sum _{k\le \lceil \delta {k}_{s}(s{\rm{^{\prime} }})\rceil }\,\sum _{l\le \lceil \delta {k}_{s{\rm{^{\prime} }}}(s{\rm{^{\prime} }}{\rm{^{\prime} }})\rceil }\,{q}_{k}{\textstyle \tfrac{k}{{N}_{k}(s{\rm{^{\prime} }})}}{q}_{l}{({\textstyle \tfrac{l}{{N}_{l}(s{\rm{^{\prime} }}{\rm{^{\prime} }})}})}^{2}\\  & \simeq  & {\textstyle \tfrac{2{s}^{2\beta }}{{d}^{2}{t}^{2\beta }}}\,{\int }_{s}^{t}\,ds{\rm{^{\prime} }}\,{\int }_{s{\rm{^{\prime} }}}^{t}\,ds{\rm{^{\prime} }}{\rm{^{\prime} }}\,{\int }^{\delta d{(\frac{s{\rm{^{\prime} }}}{s})}^{\beta }}\,dk\,{\int }^{\delta k{(\frac{s{\rm{^{\prime} }}{\rm{^{\prime} }}}{s{\rm{^{\prime} }}})}^{\beta }}\,dl{\textstyle \tfrac{\gamma -1}{\gamma s{\rm{^{\prime} }}}}{\textstyle \tfrac{\gamma -1}{\gamma s{\rm{^{\prime} }}{\rm{^{\prime} }}}}{\textstyle \tfrac{l}{s{\rm{^{\prime} }}{\rm{^{\prime} }}{\textstyle \tfrac{c}{\gamma -1}}{({\textstyle \tfrac{l}{\delta }})}^{-\gamma +1}}}\\  & = & {\textstyle \tfrac{2{s}^{2\beta }{(\gamma -1)}^{3}}{{d}^{2}{t}^{2\beta }c{\gamma }^{2}{\delta }^{\gamma -1}}}\,{\int }_{s}^{t}\,ds{\rm{^{\prime} }}\,{s{\rm{^{\prime} }}}^{-1}\,{\int }_{s{\rm{^{\prime} }}}^{t}\,ds{\rm{^{\prime} }}{\rm{^{\prime} }}\,{s{\rm{^{\prime} }}{\rm{^{\prime} }}}^{-2}\,{\int }^{\delta d{(\frac{s{\rm{^{\prime} }}}{s})}^{\beta }}\,dk\,{\int }^{\delta k{(\frac{s{\rm{^{\prime} }}{\rm{^{\prime} }}}{s{\rm{^{\prime} }}})}^{\beta }}\,dl\,{l}^{\gamma }\\  & \simeq  & {\textstyle \tfrac{D}{t}}{k}_{s}{(t)}^{\gamma },\end{array}$$

where $1\ll {k}_{s}(t) < {k}^{\ast }(t)$, and we set $k={k}_{s^{\prime} }(s^{\prime} )$, $l={k}_{{s}^{^{\prime\prime} }}(s^{\prime\prime} )$, and

$$D=\frac{2{\delta }^{\gamma +3}{(\gamma -1)}^{2}}{c(\gamma +2)\,(\gamma +1)\gamma (\,-\,1+\beta (\gamma +2))}.$$

Note that in the last line of Eq. (25), we must separately consider three cases: (i) −1 + *β*(*γ* + 1) > 0, (ii) −1 + *β*(*γ* + 1) = 0, and (iii) −1 + *β*(*γ* + 1) < 0 and −1 + *β*(*γ* + 2) > 0. When −1 + *β*(*γ* + 2) > 0, Eq. (25) can be rewritten as

$$C(k)\ge \frac{D}{t}{k}^{\gamma }.$$

## Data Availability

All the data that support our findings in this paper are available from the corresponding author upon reasonable request.
